# Free Energy Evaluation of Cavity Formation in Metastable Liquid Based on Stochastic Thermodynamics

**DOI:** 10.3390/e26080700

**Published:** 2024-08-17

**Authors:** Issei Shimizu, Mitsuhiro Matsumoto

**Affiliations:** Department of Mechanical Engineering and Science, Kyoto University, Kyoto 615-8540, Japan; matsumoto@kues.kyoto-u.ac.jp

**Keywords:** homogeneous bubble nucleation, cavitation, classical nucleation theory (CNT), nucleation free energy, critical nucleus, molecular dynamics (MD) simulation, Jarzynski equality, stochastic thermodynamics, surface tension

## Abstract

Nucleation is a fundamental and general process at the initial stage of first-order phase transition. Although various models based on the classical nucleation theory (CNT) have been proposed to explain the energetics and kinetics of nucleation, detailed understanding at nanoscale is still required. Here, in view of the homogeneous bubble nucleation, we focus on cavity formation, in which evaluation of the size dependence of free energy change is the key issue. We propose the application of a formula in stochastic thermodynamics, the Jarzynski equality, for data analysis of molecular dynamics (MD) simulation to evaluate the free energy of cavity formation. As a test case, we performed a series of MD simulations with a Lennard-Jones (LJ) fluid system. By applying an external spherical force field to equilibrated LJ liquid, we evaluated the free energy change during cavity growth as the Jarzynski’s ensemble average of required works. A fairly smooth free energy curve was obtained as a function of bubble radius in metastable liquid of mildly negative pressure conditions.

## 1. Introduction

In this research we focus on the energetics of homogeneous bubble nucleation in stretched metastable liquid. Inception of bubble nucleation is a fundamental process in the first-order liquid–vapor phase transition in general, and has been long investigated in various fields, such as cavitation [1,2], nucleate boiling [3,4], and the metastability limit of liquids [5,6,7,8], but the understanding of microscopic details are still required.

The mechanism of nucleation has been mainly studied with the classical nucleation theory (CNT) [5,6,9,10,11], which is essentially based on macroscopic thermodynamics. In the simplest form of CNT, assuming mass conservation, spherical shape of a nucleus, and a size-independent surface tension, the free energy change ΔF of the system during the growth of a bubble is given by the following equation:

(1) $$\Delta F\left(r\right)=-\frac{4}{3}\pi r^{3}\Delta f_{V}+4\pi r^{2}\gamma$$

where *r* is the bubble radius, $\Delta f_{V}$ the difference in free energy density between liquid and vapor phases, and γ the surface tension. Although the physics for Equation (1) is clear as a base of energetics of homogeneous bubble nucleation, discrepancy in the nucleation rate between CNT models and experiments is often extremely large [12], and various improvements in theoretical models have been proposed [6,11,13,14,15].

The reason for the discrepancy can be sought both in the Bolzmann factor with ΔF and the prefactor corresponding to the rate. In many macroscopic experiments in which bubble formation proceeds relatively slowly, we suppose that the macroscopic thermodynamics holds well, and thus the discrepancy should be mainly in the prefactor. On the other hand, the enegetics can be questionable in the case of rapid bubble formation (or cavitation) under extreme conditions, such as the case close to the spinodal line. In this study, we revisit the free energy change in CNT of homogeneous bubble nucleation by utilizing molecular dynamics (MD) simulations combined with stochastic thermodynamics. Several MD simulations on homogeneous bubble nucleation were reported [16,17,18,19,20]. Due to the spatial and temporal scale limits of the simulation method, however, the conditions used in these MD simulations are highly non-equilibrium, being closer to the spinodal lines, under which spontaneous bubble generation occurs within the simulation time. Furthermore, kinetics (or the nucleation rate) was the main target in these simulations while energetics is still hard to investigate because the system is in a highly non-equilibrium state during this kind of simulation.

Here, we want to investigate the free energy during the bubble nucleation. For that purpose, we adopt a basic formula of stochastic thermodynamics, with which we are able to evaluate the free energy from a special type of ensemble average of works to create and expand a bubble.

## 2. Stochastic Thermodynamics: Theoretical Background

Among several variations of free energy evaluation schemes proposed in the field of stochastic thermodynamics [21], we here adopt the simplest form, the Jarzynski equality. Consider a system contacting a thermal reservoir. Assuming any path L in the configurational space from a starting point to a goal, this equality describes a relationship between the free energy F(s) on a point s∈L (s=0 is the starting point) and the work W(s) required along the path. If we can assume that the process along the path is always quasistatic, the conventional thermodynamics tells us

(2) F(s)−F(0)=W(s)

but in general

(3) F(s)−F(0)≤W(s)

which is a representation of the second law of thermodynamics. The difference is of course dissipated, as heat *Q* in most cases,

(4) F(s)−F(0)=W(s)−Q(s)

In microscale processes where fluctuations are not negligible, *W* also contains fluctuations, and Equation (3) should be expressed as

(5) $$F\left(s\right)-F\left(0\right)\leq \langle W(s)\rangle$$

where 〈〉 represents the ensemble average.

In 1997 [22], Jarzynski showed that the following equality holds regardless of the path and the speed:

(6) $$\exp\left(-\frac{F(s)-F(0)}{k_{B}T}\right)=\langle \exp\left(-\frac{W(s)}{k_{B}T}\right)\rangle$$

where *T* is the temperature of thermal reservoir, and $k_{B}$ is the Boltzmann constant. This equality only requires that the system is initially in equilibrium. The point in Equation (6) is that we can evaluate the state quantity F(s) without the conventional assumption of quasistatic process. This gives a great merit for us to evaluate free energy with MD simulations because a typical time scale of MD simulations is so short that, in many cases, we cannot assume thermal equilibrium however slow the system change is.

In this work, we targeted simple liquid and conducted a series of MD simulations to observe “bubble” generation by applying an external force. Then, by adopting Equation (6), the free energy change during the bubble growth was evaluated.

The applied field forces all particles out of the bubble so that its inside is always a vacuum. In this sense, our investigation is for the energetics of cavity formation, not the generation of vapor bubbles. In the (n,v) coodinate space [23], where *n* is the number of vapor particles in the bubble of volume *v*, this corresponds to the change along n=0 path. For more general cases in (n,v) space, some modification may be possible, which is briefly discussed in Section 5.5.

## 3. System and Method

We carried out a series of MD simulations to investigate the free energy for a bubble of several nanometers in monatomic liquid. We focus on this scale of bubbles, because the bubble nucleation often initiates from this scale of cavity. It is still hard to experimentally observe bubbles of this scale, and much computer resource is required to investigate them with conventional molecular simulations. All MD simulations were executed with LAMMPS [24,25]. OVITO [26] was utilized to visualize the atomic configurations.

### 3.1. Simulation System and Procedure

We adopted the Lennard-Jones (LJ) 12-6 potential as the particle–particle interaction,

(7) $$\phi \left(r\right)=4\varepsilon \left[{\left(\frac{\sigma }{r}\right)}^{12}-{\left(\frac{\sigma }{r}\right)}^{6}\right],$$

where *r* is the particle–particle distance. ε and σ are the parameters for energy and length, respectively. The interaction is truncated at $r=r_{c}=3.5\;\sigma$ to save the computational resources. Dependence of results on $r_{c}$ is briefly discussed in Section 5.1. In the following descriptions, all quantities are expressed with the conventional reduced units, such as ε: energy, σ: length, and *m*: particle mass. Just for reference, typical values for argon [27] are $\varepsilon ≃1.66\times 10^{-21}$ J, $\sigma ≃0.34\times 10^{-9}$ m, and the time unit $\tau \equiv \sigma \sqrt{m/\varepsilon }≃2.15\times 10^{-12}$ s. All MD simulations were executed with time step 0.002τ. Periodic boundary conditions are assumed for all directions.

With conventional isothermal–isobaric (NPT-constant) ensemble conditions, where the temperature is controlled with the Nosé–Hoover thermostat [28] and the pressure with the Martyna barostat [29], we performed a series of MD simulations for cavitation under external force and evaluated the free energy change based on the Jarzynski formula, Equation (6). Each simulation consists of three steps:

1. Liquid in equilibrium at given temperature $T_{0}$ and pressure $p_{0}$ is prepared using a standard procedure of MD simulation.
2. A spherical force field $\phi_{B}$ at the center of the system box is applied for all LJ particles, and the system is equilibrated again at $\left(T_{0},p_{0}\right)$. We assume an LJ-type field as
  (8) $$\phi_{B}\left(r_{o},t=0\right)=4\varepsilon \left[{\left(\frac{\sigma }{r_{o}}\right)}^{12}-{\left(\frac{\sigma }{r_{o}}\right)}^{6}\right],$$
  where $r_{o}$ is the distance of each particle from the box center. The field is also truncated at $r_{o}=r_{c}=3.5\;\sigma$.
3. For the main simulation, the radius of the force field is increased and the resulting “bubble” growth is monitored.
  (9) $$\phi_{B}\left(r_{o},t\right)=4\varepsilon \left[{\left(\frac{\sigma }{r_{o}-R\left(t\right)}\right)}^{12}-{\left(\frac{\sigma }{r_{o}-R\left(t\right)}\right)}^{6}\right],$$
  where R(t) is a time-dependent variable corresponding to the bubble radius, where the bubble is the region of excluding LJ particles. In this paper, we simply adopted $\phi_{B}$ with a size linearly increasing with time,
  (10) $$R\left(t\right)=v_{0}t$$
  where $v_{0}$ is a positive constant. $v_{0}$ dependence is briefly discussed in Section 5.4.

During the MD calculation, atomic data (position ${\vec{r}}_{i}$, velocity ${\vec{v}}_{i}$, and the exerted external force ${\vec{f}}_{i}\equiv -\partial \phi_{B}/\partial {\vec{r}}_{i}$) for each LJ particle *i* are stored every *k* steps for later analysis.

To apply the Jarzynski equality to our system for the free energy evaluation, a large number of samplings are required; for that purpose, we took the ensemble average over 100 different atomic configurations at equilibrium as the initial conditions.

**Figure 1:**
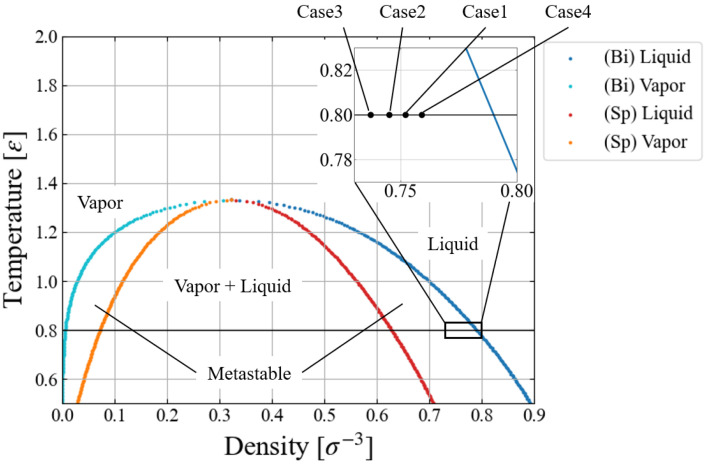
Simulation conditions plotted in a liquid–vapor phase diagram of LJ fluid based on an empirical equation of states [32]. Blue and cyan symbols: binodal lines, red and orange: spinodal (stability limit) lines.

The simulation conditions are summarized in Table 1. We chose a relatively low temperature ($T_{0}=0.8$) since the inside of the bubble (or cavity) is always a vacuum for this free energy evaluation while applying the external force field. Four different environmental pressure conditions, $-0.30\leq p_{0}\leq -0.15$, are investigated to see how $p_{0}$ affects the nucleation behavior. In view of the standard phase diagram and the equation of states for LJ fluid [30,31,32], these pressure conditions are extremely mild, i.e., much closer to the binodal line than to the spinodal one, as shown in Figure 1. This is the region difficult for conventional MD simulations to access [16,17,18,33] due to the large activation energy. Since a large size of critical nucleus was expected for the largest pressure condition (Case 4), we prepared a larger system with 108,000 particles to mitigate the artifact of the finite system size. The growth speed $v_{0}=0.01$ corresponds to 1.6 m/s in argon units, which is still very fast; the speed dependence of results is briefly discussed in Section 5.4. For the larger system, Case 4, we adopted an even larger speed $v_{0}=0.02$ to save the computation time.

### 3.2. Free Energy Evaluation

On completing the MD simulations for each Case, we adopted the Jarzynski equality, Equation (6), to evaluate the free energy change during the bubble growth with the following steps:

Step 1: The work during a short period from time *t* and t+Δt is evaluated as
  (11) $$w\left(t\right)=\sum_{i}{\vec{f}}_{i}\left(t\right)\cdot {\vec{v}}_{i}\left(t\right)\Delta t$$
  In this simulation, we chose Δt=5MDsteps=0.01 [τ].
Step 2: The accumulated work, which is the sum of the stepwise work up to time t=nΔt, is determined.
  (12) $$W\left(t\right)=\sum_{n^{\prime }=0}^{n}w\left(n^{\prime }\Delta t\right)$$
Step 3: We perform simulations multiple times, calculating W(t) for each case. The free energy change ΔF(t) from the initial state is evaluated by taking the ensemble average as the Jarzynski equality, Equation (6), as a function of *t*.
Step 4: Since the bubble “radius” is directly related to time *t* with Equation (10), we finally obtain the free energy as a function of bubble radius.

## 4. Results

### 4.1. Thermodynamic Properties

**Figure 2:**
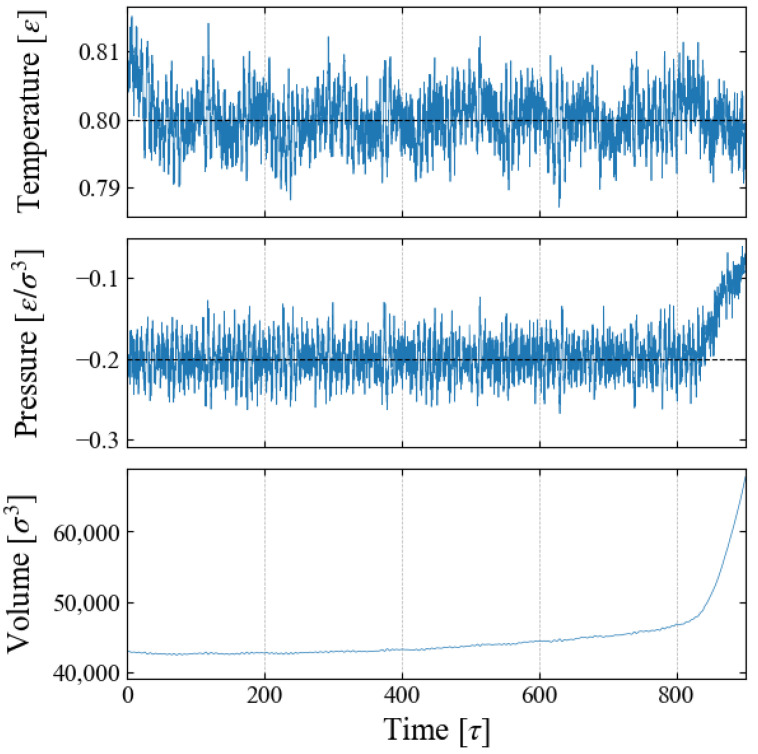
Property change during a simulation; example for Case 1 ($p_{0}=-0.20$).

We carried out NPT ensemble MD simulations for the main sampling, during which the system volume *V* changes due to the time-varying external field $\phi_{B}\left(t\right)$. An example is shown in Figure 2 for Case 1, indicating that the barostat to control the system pressure fails after time *t*∼800 [τ]. This is caused by spontaneous bubble growth, as discussed below.

### 4.2. Bubble Growth

**Figure 3:**
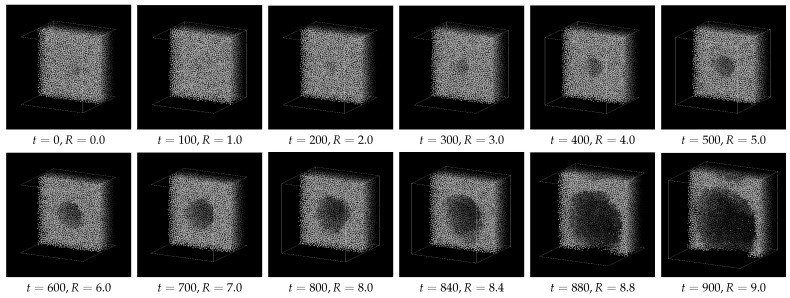
Examples of cross-sectional view for Case 1 ($p_{0}=-0.20$).

Figure 3 indicates a series of snapshots (cross-sectional views) for Case 1. The “bubble” generated by the external field $\phi_{B}$ gradually grows up to t≃700; it seems to become unstable at *t*∼800, where its shape is distorted and the growth is accelerated, and finally the thin liquid film between the neighboring bubbles is broken due to the periodic boundary conditions.

**Figure 4:**
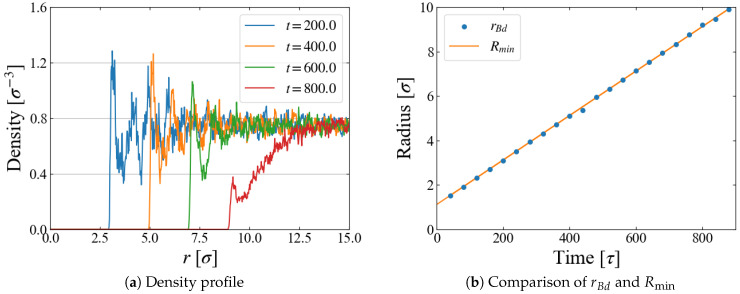
(**a**) Density profile, the first peak of which defines the bubble radius $r_{Bd}$, (**b**) change of $r_{Bd}$ compared with the position of external field minimum $R_{\min}$. Example data for Case 1.

A quantity relevant to the nucleation analysis is the bubble radius at time *t*, and we evaluate it in two ways here. One is based on the density profile of LJ particles. An example for Case 1 ($p_{0}=-0.20$) is shown in Figure 4a, where the number density is plotted as a function of the distance *r* from the box center, i.e., the center of the external field $\phi_{B}$. The first peak indicates the particles situated on the “bubble surface”, from which we define the bubble radius $r_{Bd}$. As the size *R* of $\phi_{B}$ increases with time, Equation (10), the position of the first peak, shifts to larger *r* accordingly. We expect that $r_{Bd}$ is close to the position of $\phi_{B}$ minimum, $R_{\min}\left(t\right)\equiv R\left(t\right)+2^{1/6}\sigma$; comparison is made in Figure 4b, which shows they are almost identical. In the density profile, it is still possible to see the first peak after the bubble becomes unstable at t≥800 [τ], but the peak is very small and the density in its vicinity is much less than the bulk liquid one, which suggests that the first peak at this stage is brought by weak “adsorption” on, or entrapment by, the external field.

The change in the total volume of the system V(t) during the simulation, an example of which is shown in Figure 2, provides another way to define the bubble radius. We assume that the density of “bulk” liquid is constant, which is apparent in Figure 4a. Then the change of V(t) is brought only by the bubble growth, and we define and evaluate the bubble radius $r_{Bv}$ with the spherical bubble assumption.

**Figure 5:**
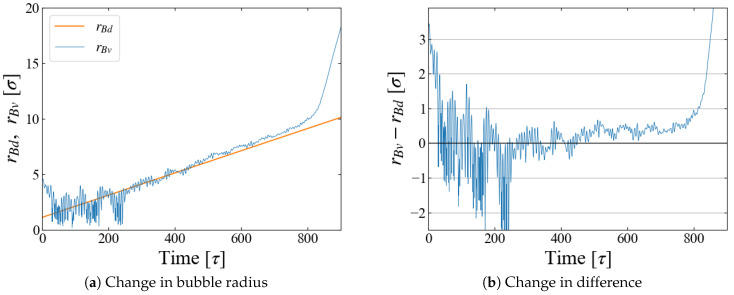
Comparison of bubble radius $r_{Bd}$ and $r_{Bv}$; example data for Case 1.

The obtained $r_{Bv}$ is compared with $r_{Bd}$ in Figure 5. The fluctuations in box size bring the large fluctuations in $r_{Bv}$, especially at the initial stage of t≤300 [τ], but the agreement of $r_{Bd}$ and $r_{Bv}$ is reasonable for t≤700 [τ], as seen in Figure 5b. Based on these data, we adopt a rather arbitrarily determined criterion in the following data analyses that the spontaneous bubble growth starts when the difference $r_{Bv}-r_{Bd}$ exceeds 1σ; in this time region, the application of the Jarzynski’s scheme fails because the “work” may not be properly evaluated with Equation (11).

### 4.3. Work and Free Energy

**Figure 6:**
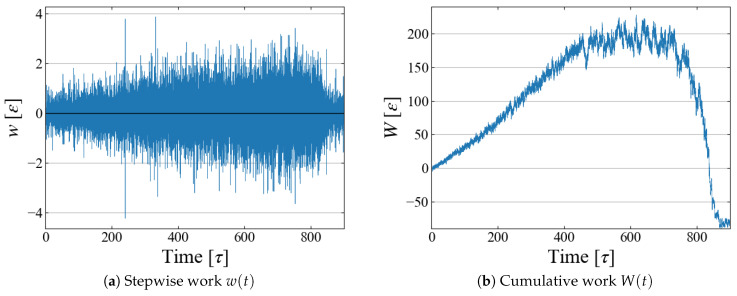
Obtained work as a function of time; example data for Case 1.

**Figure 7:**
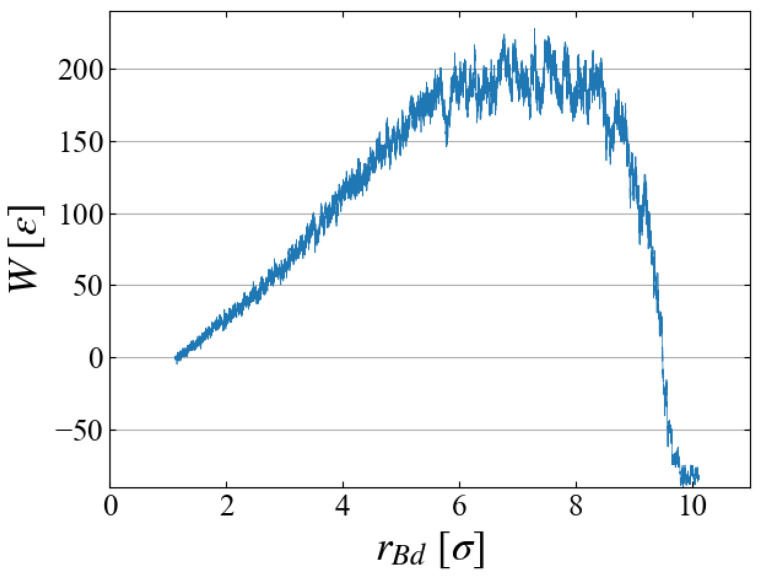
Cumulative work as a function of bubble radius; example for Case 1.

Example data of the stepwise work w(t) and cumulative one W(t) are shown in Figure 6; although fluctuations are large in w(t), their running sum W(t) seems to have a reasonable shape as expected in typical nucleation processes. By utilizing the relation between $r_{Bd}$ and *t* as in Figure 4b, the cumulative work *W* is plotted as a function of bubble radius $r_{Bd}$ in Figure 7, which illustrates the work required to generate a bubble of size $r_{Bd}$. As expected in CNT, $W(r_{Bd})$ increases up to some “critical size”; bubbles larger than this size spontaneously grow, leading to negative *W*.

**Figure 8:**
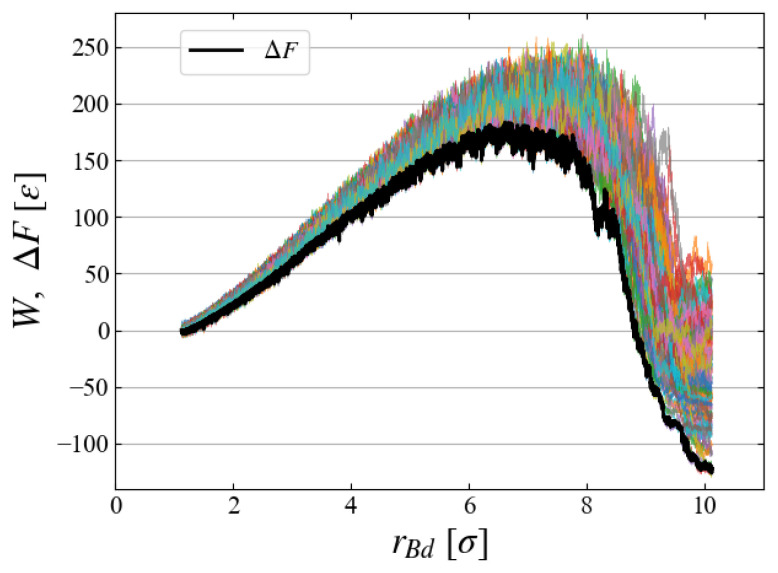
(Color curves) 100 samples of cumulative *W*, (Black) evaluated free energy difference ΔF; example for Case 1.

To evaluate the free energy change, we carried out 100 MD simulations starting from different initial configurations, each of which gives a different $W(r_{Bd})$ curve. All curves are superimposed in Figure 8, indicating large fluctuations of 50∼100 [ε]. The ensemble average based on Equation (6) gives a relatively smooth curve of free energy difference:

(13) $$\Delta F\left(r_{Bd}\right)\equiv F\left(r_{Bd}\left(t\right)\right)-F\left(r_{Bd}\left(t=0\right)\right)$$

Note that the applied external field $\phi_{B}$, Equations (9) and (10), has a finite size even at t=0, thus $\Delta F(r_{Bd})$ does not start from the origin but from $r_{Bd}≃2^{1/6}$ [σ]. Figure 8 clearly indicates the existence of a critical nucleus at ≃6.5 [σ] with activation energy 190±10 [ε]. It is apparent that the Jarzynski equality makes the ΔF curve very close to the data of minimum *W*.

**Figure 9:**
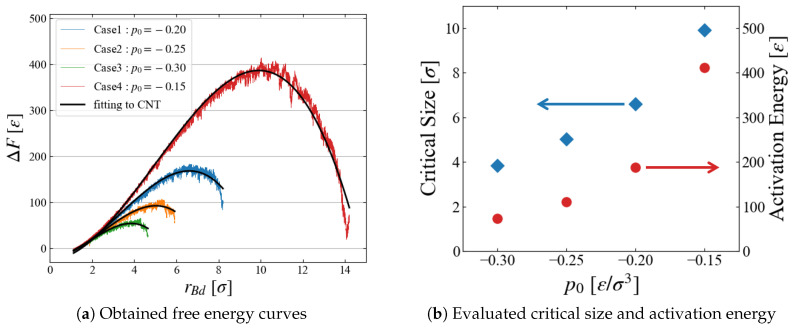
(**a**) Obtained free energy ΔF and fitting to a simple CNT as discussed in Section 5.2. (**b**) Critical bubble size and activation energy.

The results of $\Delta F(r_{Bd}$) are summarized for all four cases of different pressure conditions in Figure 9a. We successfully obtained a smooth curve for each condition; as expected, the critical bubble size and the energy barrier reduces as $p_{0}$ becomes more negative. The estimated critical size and the activation energy are shown in Figure 9b; the dependence on the surrounding pressure $p_{0}$ seems reasonable.

The evaluated activation energy is very large because we investigated systems under mild $p_{0}$ conditions. For example, the obtained energy is about 400 [ε] at $p_{0}=-0.15$, leading to an extremely small Boltzmann factor $\exp\left(-400/0.8\right)∼10^{-216}$, which is not accessible with conventional molecular simulation techniques.

## 5. Discussion

### 5.1. Critical Size

Although many theoretical and simulational investigations on bubble nucleation exist, it is still hard to estimate the critical bubble size under various metastable conditions. By adopting NPT ensemble MD simulations, Torabi et al. [23] evaluated the critical bubble size based on the stability of a spherical cavity with given size in metastable LJ liquid. Their conditions (T=0.8 and density ρ=0.745) are coincidently the same as Case 2 of our system, which enables us to make comparison. They reported the critical size of 3.2σ, which is slightly smaller than our result of ∼5σ. We suppose that this is caused by the difference in the cutoff radius $r_{c}$. They adopted a larger value of $r_{c}=4.0\;\sigma$ than ours of $r_{c}=3.5\;\sigma$, which brings more negative pressure (i.e., closer to the spinodal line) of $p=-0.33\;[\varepsilon /\sigma^{3}]$ than the −0.25 of our system (Case 2).

Kwak et al. [34] investigated the bubble nucleation kinetics with an atomic interaction model, discussing the critical size and the stability limit (tensile strength) of Ar; the comparison is not straightforward due to the difference in temperature condition, but the predicted critical size of ∼5.8[σ] gives reasonable agreement.

### 5.2. Comparison with CNT

**Figure 10:**
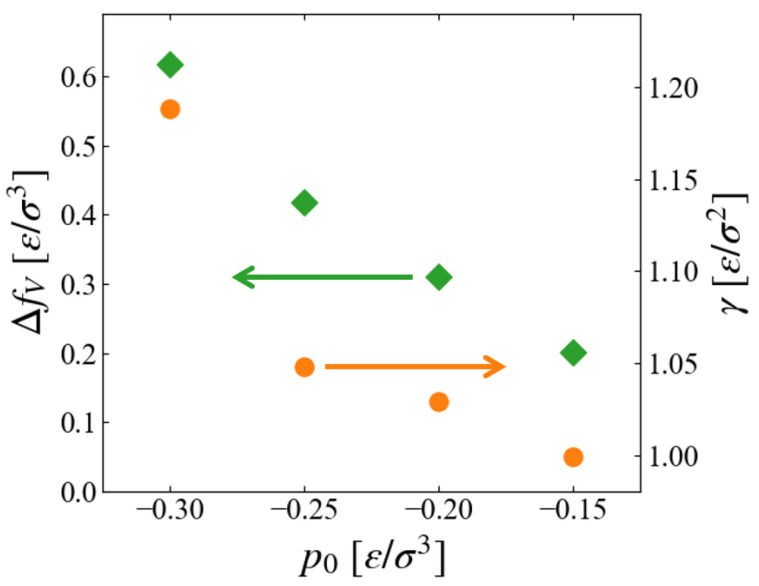
Parameters of CNT depending on the environment pressure $p_{0}$; (green) free energy density difference $\Delta f_{V}$, (orange) surface tension γ.

Here, we only make comparison with the simplest form of CNT. The obtained $\Delta F(r_{Bd})$ data are fitted to a simple equation by the least squares method,

(14) $$\Delta F\left(r\right)=ar^{3}+br^{2}+c$$

where *a*, *b*, and *c* are the fitting parameters. In the simplest CNT as shown in Equation (1), *a* and *b* are related to the free energy density difference between the bulk liquid and bulk vapor, $\Delta f_{V}\equiv f_{\mathrm{liq}}-f_{\mathrm{vap}}$, and the surface tension γ, respectively. As Figure 9a indicates, the obtained free energy curves are approximated well by Equation (14). Plotted in Figure 10 are the $p_{0}$ dependence of extracted values,

(15) $$\Delta f_{V}=\frac{a}{4\pi /3}$$

and

(16) $$\gamma =\frac{b}{4\pi }$$

Since the inside of the generated “bubble” is vacuum in this scheme, $\Delta f_{V}$ should correspond to the free energy density of expanded liquid, and the results seem reasonable that $f_{V}$ of liquid increases for larger metastability, or negatively larger $p_{0}$.

The pressure dependence of surface tension is worth noting. As far as we have noticed, no data for surface tension (interfacial tension between liquid and vapor) in non-equilibrium states have been reported, although a number of studies exist for its curvature dependence for nano-bubbles [35,36,37,38]. For equilibrium LJ fluid at T=0.8, γ≃0.8−0.9 [$\varepsilon /\sigma^{2}$] is often referred [31]. Figure 10 suggests that γ, or the surface excess free energy, is an increasing function of the “degree of non-equilibrium,” which seems reasonable; the evaluation of its accuracy is left for future investigation.

### 5.3. Sampling

**Figure 11:**
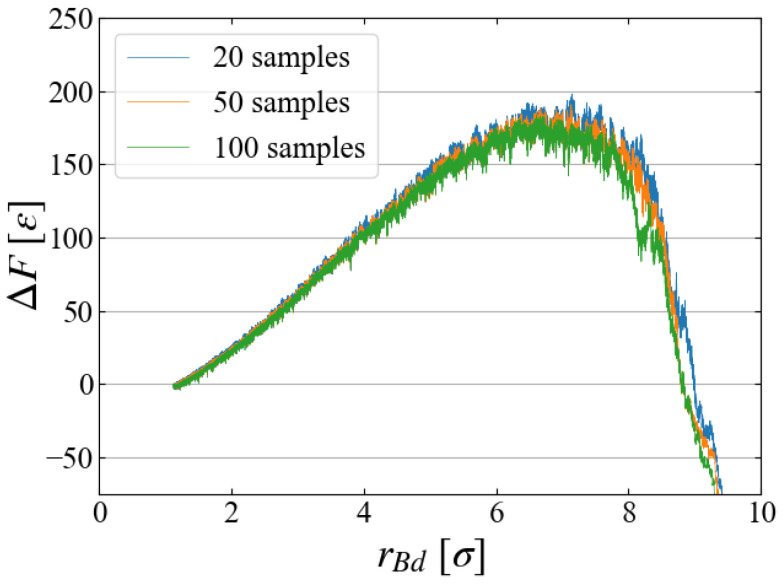
Comparison of ΔF with different number of samples for Case 1.

As the main part of free energy evaluation, we used 100 samples for each Case. To see if 100 samples are sufficient or not, we compared the evaluated ΔF in Figure 11 for Case 1, where the results using the first 20, the first 50, and all 100 samples are plotted. The difference in the averaged values seems negligibly small, and we may conclude that 100 samples are sufficient. However, a closer look at the distribution of work may give a different view.

**Figure 12:**
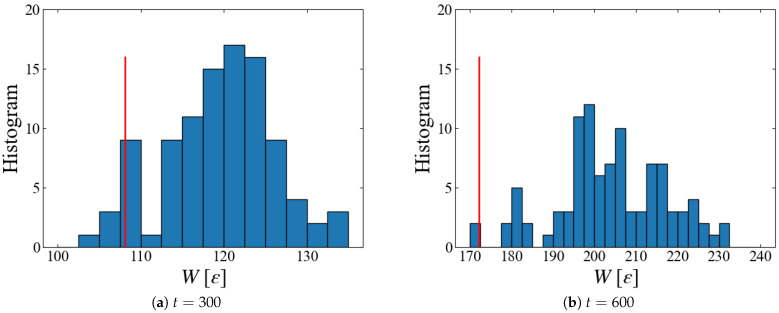
Distribution of accumulated work W(t): red line indicates the free energy estimated with Equation (6); examples for Case 1.

Plotted in Figure 12 are histograms of the accumulated work W(t) at different times. Since data with smaller *W* have larger weights in Jarzynski’s average, Equation (6), we need a sufficient number of samples in the region of smaller *W*. However, we have only four samples at t=300 and two at t=600 that are smaller than the obtained ΔF, suggesting that 100 samples may not be sufficient. Thus, a further increase in the number of samples may significantly lower the free energy.

### 5.4. Growth Speed

**Figure 13:**
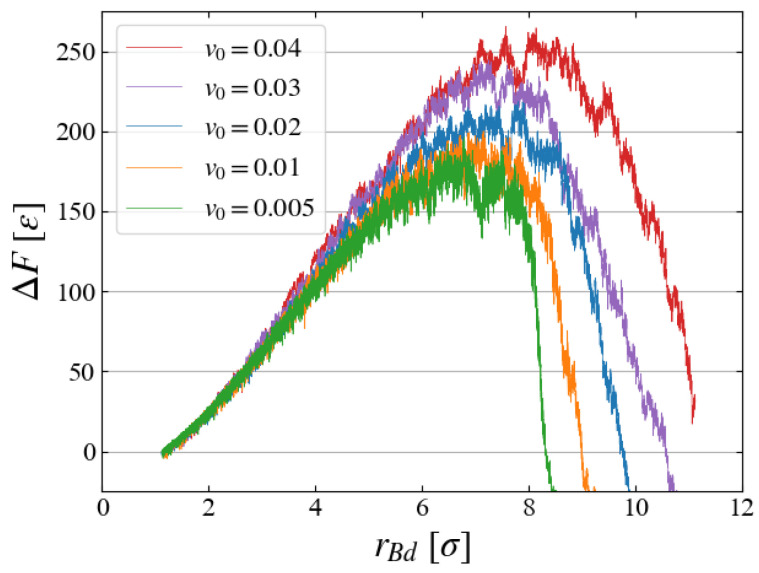
$v_{0}$ dependence of ΔF for systems with $p_{0}=-0.20$; average is taken over ten samples as this preliminary evaluation.

We briefly discuss how the bubble growth speed $v_{0}$ affects the evaluation of ΔF. In preliminary investigations, five cases with different $v_{0}$ ($0.005\leq v_{0}\leq 0.04$) are compared, as in Figure 13, where ΔF is evaluated with Equation (6) with ten samples for each case. The free energy increases for larger expanding speeds, probably because the liquid structure is not sufficiently relaxed for faster bubble expansion. However, the results for $v_{0}=0.01$ and 0.005 look similar, and thus we have chosen $v_{0}=0.01$ for the main simulations.

### 5.5. Relation to Bubble Formation

**Figure 14:**
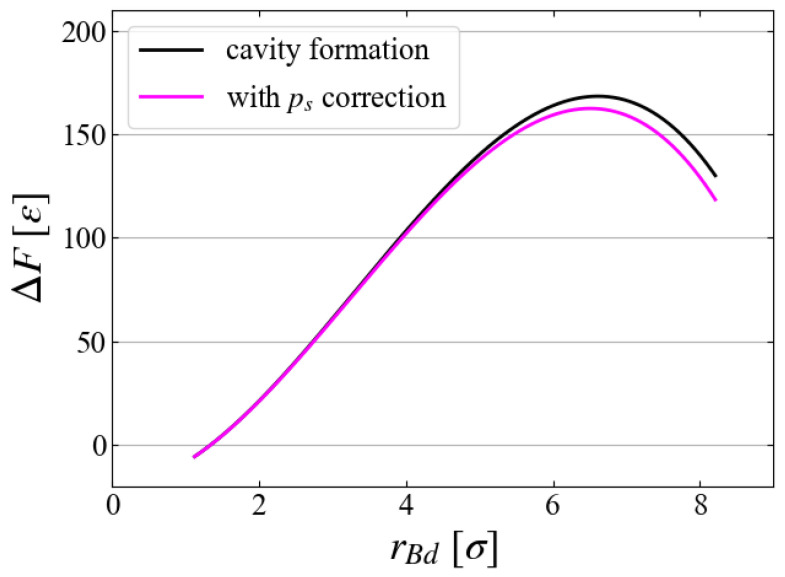
Vapor pressure correction to the cavity formation free energy; example for Case 1.

Finally, we discuss the relation of our results to bubble formation. Although we have evaluated the free energy of a cavity, inside of which is a vacuum, we can make a simple correction for a vapor bubble by considering vapor pressure as

(17) $$\Delta F_{cor}=\Delta F-\frac{4\pi }{3}{r_{Bd}}^{3}p_{v}$$

where $p_{v}$ is the vapor pressure inside a bubble of the same size as our cavity. As an approximation, here we adopt the saturated vapor pressure $p_{s}=0.005$ at T=0.8 [32] as $p_{v}$, although its curvature dependence is well-known [39]. Figure 14 indicates that the correction is small in general under this low temperature condition, ∼6ε near the critical size. This difference can be even less, by considering the curvature dependence and the transient effect (retarded evaporation in a rapidly growing bubble). Free energy change along another practical path in (n,v) space lies between these.

## 6. Conclusions

Using a standard molecular dynamics (MD) simulation technique, we investigated the free energy change during homogeneous bubble nucleation based on a stochastic thermodynamics formulation, namely, the Jarzynski equality, for a simple Lennard-Jones fluid system with an expanding external force field. It is shown that, when compared with the direct application of conventional MD simulations for various types of nucleation processes, we can evaluate the free energy under much milder (i.e., closer to the binodal lines) conditions. Yet, assumption of thermal equilibrium is not required for free energy evaluation, in contrast to the often-adopted molecular simulations for thermal properties. It should be noted, however, sufficient number of samplings are essential, and may require much computational resources.

In this work, we adopted simple liquid to see applicability of the Jarzynski equality, but the here-developed scheme can be used for various other liquids. Investigations on water and aqueous electrolyte solutions are under way.

One technical restriction still exists for this method, which is the assumption of spherical bubble shape. It becomes questionable at higher temperatures, so application of this method is currently limited only to low-temperature conditions.

Since the establishment of the Jarzynski equality, several extensions and generalizations have been proposed [21,40,41]. For example, the Crooks fluctuation theorem [42] relates the probability distributions of the forward process and the backward one. These extended methods may provide a better tool to investigate bubble nucleation, which is left for future study.

## Figures and Tables

**Table 1:** Conditions for the main simulation.

	Number of Particles N [–]	Temperature $T_{0}$ [ε]	Pressure $p_{0}$ [$\varepsilon /\sigma^{3}$]	Equilibrium Density ρ [$\sigma^{-3}$]	Growth Speed $v_{0}$ [σ/τ]	Number of Samplings [–]
Case 1	32,000	0.80	−0.20	0.752	0.01	100
Case 2	32,000	0.80	−0.25	0.745	0.01	100
Case 3	32,000	0.80	−0.30	0.737	0.01	100
Case 4	108,000	0.80	−0.15	0.759	0.02	100

## Data Availability

Data are available from the authors upon request.
